# Transcriptional response to mild therapeutic hypothermia in noise-induced cochlear injury

**DOI:** 10.3389/fnins.2023.1296475

**Published:** 2024-01-17

**Authors:** Samantha Rincon Sabatino, Rachele Sangaletti, Anthony Griswold, W. Dalton Dietrich, Curtis S. King, Suhrud M. Rajguru

**Affiliations:** ^1^Department of Biomedical Engineering, University of Miami, Coral Gables, FL, United States; ^2^Department of Otolaryngology, University of Miami, Coral Gables, FL, United States; ^3^Department of Human Genetics, University of Miami, Coral Gables, FL, United States; ^4^The Miami Project to Cure Paralysis, University of Miami, Coral Gables, FL, United States; ^5^RestorEar Devices LLC, Bozeman, MT, United States

**Keywords:** noise-induced hearing loss, hidden hearing loss, therapeutic hypothermia, hair cells, transcriptional, mechanisms, inflammation, cytokines

## Abstract

**Introduction:**

Prevention or treatment for acoustic injury has been met with many translational challenges, resulting in the absence of FDA-approved interventions. Localized hypothermia following noise exposure mitigates acute cochlear injury and may serve as a potential avenue for therapeutic approaches. However, the mechanisms by which hypothermia results in therapeutic improvements are poorly understood.

**Methods:**

This study performs the transcriptomic analysis of cochleae from juvenile rats that experienced noise-induced hearing loss (NIHL) followed by hypothermia or control normothermia treatment.

**Results:**

Differential gene expression results from RNA sequencing at 24 h post-exposure to noise suggest that NIHL alone results in increased inflammatory and immune defense responses, involving complement activation and cytokine-mediated signaling. Hypothermia treatment post-noise, in turn, may mitigate the acute inflammatory response.

**Discussion:**

This study provides a framework for future research to optimize hypothermic intervention for ameliorating hearing loss and suggests additional pathways that could be targeted for NIHL therapeutic intervention.

## 1 Introduction

Acoustic injury produces a multifaceted response in the auditory system that corresponds to the extent and nature of the noise insult. Given the lack of FDA-approved otoprotectants, the time course of noise-induced mechanical and molecular injury and repair in the cochlea is being increasingly studied to identify viable otoprotective or therapeutic interventions for one of the most common forms of acquired hearing loss, i.e., noise-induced hearing loss (NIHL).

Acute noise-induced peripheral pathology includes mechanical damage to structures within the organ of Corti as well as alterations to cochlear metabolism and microcirculation, which may, in turn, induce lasting secondary effects. Studies have reported decreased cochlear blood flow (Okamoto et al., 1992; Arpornchayanon et al., 2011), generation of reactive oxygen species (Ohlemiller et al., 1999), and increased calcium release (Maurer et al., 1993; Fridberger et al., 1998) in the cochlea within the first 48 h post-noise. The noise-induced vasoconstriction and cochlear reperfusion injury produce metabolic stress within the cochlea, leading to increased superoxide radicals following noise exposure (Yamashita et al., 2004; Sai et al., 2020). Noise-induced changes in calcium release and accumulation have also been documented with an early increase in the acute recovery period and a chronic accumulation in outer hair cells and cochlear endolymph observed for several days post-noise (Ikeda et al., 1988; Maurer et al., 1993). This increase in accumulated free calcium is suggested to precede noise-induced glutamate excitotoxicity and possible synaptopathy or neuropathy (Bing et al., 2015; Sebe et al., 2017; Wang et al., 2018; Liu et al., 2020). Several studies have also identified regulation of the immune and inflammatory response in the early development of NIHL (Cai et al., 2014; Vethanayagam et al., 2016; Yang et al., 2016; Frye et al., 2019), including macrophage and neutrophil recruitment in the cochlea following acoustic injury (Hirose et al., 2005; Tornabene et al., 2006; Yang et al., 2015; Rai et al., 2020; Shin et al., 2022).

Evaluation of differential gene expression following acoustic insult has shed light on targets relevant to both the acoustic injury and subsequent repair processes. Investigations employing gene microarrays and polymerase chain reaction (PCR) pinpointed regulatory genes associated with inflammation, apoptosis, early transcriptional activity, and stress response (Lomax et al., 2001; Taggart et al., 2001; Cho et al., 2004; Kirkegaard et al., 2006; Hu et al., 2009; Gratton et al., 2011; Han et al., 2012). Notably, studies using knockout or knockdown models have identified the role of genes, such as Sod1 (Ohlemiller et al., 1999), Gpx1 (Ohlemiller et al., 2000), Vasp (Schick et al., 2004), Trpv4 (Tabuchi et al., 2005), Hsf1 (Fairfield et al., 2005), P2rx2 (Yan et al., 2013), Nox3 (Lavinsky et al., 2015), Nrf2 (Honkura et al., 2016), and A1ar (Vlajkovic et al., 2017). With advancements in RNA sequencing, a more comprehensive analysis of transcriptional changes immediately following cochlear damage caused by noise has become possible. This technique has been instrumental in elucidating the molecular events within the ear following different types of acoustic insults, including blast trauma (Wang Y. et al., 2020) and noise exposures producing either permanent or temporary threshold shifts (PTS, TTS; Patel et al., 2013; Yang et al., 2015, 2016; Maeda et al., 2017; Wei et al., 2020; Bae et al., 2021; Milon et al., 2021; Warnecke et al., 2021). These studies have identified distinct regulation of the inflammatory response, with particular emphasis on cytokine/chemokine signaling, toll-receptor signaling, and complement and coagulation cascades (<48 h). A combined study using both RiboTag and single-cell RNA sequencing has constructed a location and temporal map of acoustic trauma at multiple timepoints up to 7 days post-exposure to a PTS-inducing noise (Milon et al., 2021). The study demonstrated enrichment of apoptotic signaling, innate immune response, and cytokine response primarily in outer hair cells and supporting cells. NIHL involves a complex interplay of oxidative stress, inflammatory responses, and mechanisms that damage to the delicate structures within the inner ear.

Previous research has shown that both systemic and local cooling can protect cochlear structures, limiting damage from noise exposure (Henry, 1980; Berndt and Wagner, 1981; Henry and Chole, 1984; Henry, 2003), cisplatin-induced injury (Spankovich et al., 2016; Stanford et al., 2020), and electrode-induced trauma (Balkany et al., 2005; Tamames et al., 2016; Sangaletti et al., 2022). Therapeutic hypothermia is a complex intervention that may engage a variety of pathways for protection, such as limiting oxidative stress damage and the accumulation of calcium and glutamate, thereby reducing excitotoxicity and mitigating inflammation and apoptosis (Sangaletti et al., 2022). The modulatory effects of hypothermia may also include the downregulation of transcription factors, such as c-Fos and JunB, and several cytokines (Ohta et al., 2007; Shi et al., 2017). Temperature modulation also regulates the activity of Sod1, Vasp., Trpv4, Hsf1, and Nrf2 for neuroprotection in animal models (Nagel et al., 2012; Kaija et al., 2014; Kida et al., 2014; Salman et al., 2017; Yan et al., 2022). The activation of cold shock proteins, such as rescue gene RBM3, which is synthesized in response to hypothermia, may also confer methods of protection against hypoxic injury and NMDA glutamate receptor activation.

In a companion study (Rincon Sabatino et al., 2023), we demonstrate therapeutic benefits of targeted hypothermia following noise exposure. In rats, localized temperature management of the inner ear protects residual hearing shortly following noise exposure, and hypothermia promotes maintenance of synaptic contacts. As the hypothermic intervention is performed following cessation of noise exposure, hypothermia may limit early noise-induced mechanical damage, ROS production, calcium release, and glutamate excitotoxicity, contributing to lower temporary shifts in hearing. However, given the acute recovery observed within 24 h post-exposure, we hypothesize that secondary changes in inflammatory genes will be primarily dysregulated between treated and untreated animals. Here, we perform transcriptional profiling using the validated hypothermia paradigm to determine the molecular pathways of protection involved in the hypothermic mitigation of NIHL in male rats, at a timepoint in which we demonstrated a clear beneficial effect (Rincon Sabatino et al., 2023).

## 2 Materials and methods

### 2.1 Experimental design

**Figure 1:**
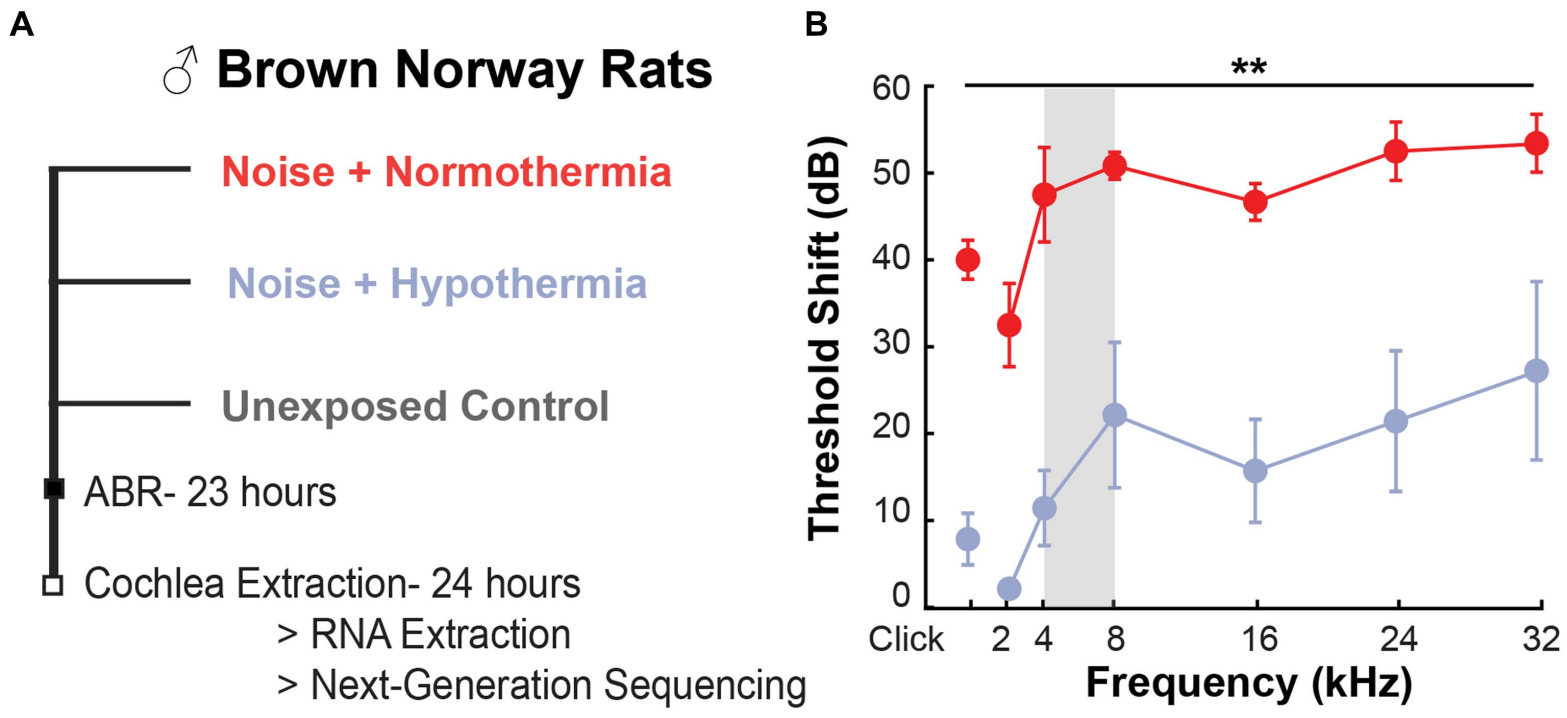
ABR threshold preservation with hypothermia following acute noise exposure. **(A)** Experimental design used for post-noise hypothermia and normothermia TTM. **(B)** Obtained ABR threshold shift from pre-noise measurements at 23 h post-exposure to 105 dB noise (2 h, 4–8 kHz, vertical gray bar) in hypothermia and normothermia TTM-treated animals (*n* = 3/group, male Brown Norway rat). The unexposed controls had no shift in thresholds within 24 h.

All procedures were approved by the University of Miami Animal Care and Use Committee. Male Brown Norway rats aged 15–17 weeks old (225–250 grams) were randomly separated into three groups: *Noise + Normothermia*, *Noise + Hypothermia*, and *Unexposed Control*. As described in the companion study (Rincon Sabatino et al., 2023), noise-exposed groups received 2 h of continuous noise at 105 dB SPL under isoflurane anesthesia followed by 2 h of targeted temperature management (TTM) under anesthesia (ketamine/xylazine, 44/5 mg/kg). Auditory brainstem responses (ABRs) were recorded prior to noise to establish baseline and at approximately 24 h post-noise. Cochleae were immediately extracted for RNA isolation (Figure 1A).

### 2.2 Hearing tests

Auditory brainstem response (ABR) tests were performed in anesthetized animals (ketamine/xylazine, 44/5 mg/kg) to determine hearing thresholds in each ear for pure tones between 2 and 32 kHz and click stimulus. Baseline hearing was similar across the groups (two-way ANOVA, frequency*treatment, *p* = 0.3870). Physiological temperature during ABRs was maintained with heating pads and monitored with rectal temperature probes. The ABR tests were conducted in a soundproof chamber; acoustic stimuli were delivered through speakers placed within auditory ear canals; and responses were measured with subcutaneous recording electrodes placed behind each ear, reference electrodes at the skull vertex, and a grounding electrode above the muscle in the hind leg. Responses were recorded with the Intelligent Hearing Systems data acquisition system (IHS, SmartEP). System settings were previously described (Tamames et al., 2016), with the calibration of the high- and low-frequency transducers performed for all tested intensities and frequencies in the Brown Norway rat model. In brief, ABRs were averaged at each frequency over 1,024 sweeps of 1-ms stimulus. ABRs were collected in 10 dB SPL steps, decreasing from 80 dB SPL. A recognizable Wave I peak was the minimum criteria for determining the threshold. If ABR Wave 1 was not identified at the 80 dB maximal intensity limit, the threshold of the ear and frequency was set at 90 dB for estimates of the threshold shift. Threshold shifts were determined by subtracting the ear- and frequency-specific pre- and post-noise thresholds. Threshold shifts were averaged for both ears corresponding to a single animal.

### 2.3 Noise exposure

Rats were maintained under anesthesia (1.5–2% isoflurane and 1% oxygen) for the duration of noise exposure duration (2 h) in a soundproof chamber. Physiological temperatures were continuously monitored and regulated with far infrared warming pads to maintain stable rectal temperature at 37°C (RightTemp Jr., Kent Scientific). Animals were exposed to a continuous 2-h monaural narrowband noise ranging from 4 to 8 kHz from an overhead speaker (Pyle PDBT45) driven by an amplifier (Pyle PPA450). Noise was recorded every 15-min intervals to ensure 105 dB SPL exposure to both ears (SPL meters, UT353 BT). Following noise exposure, animals were observed until fully awake after isoflurane exposure before proceeding to targeted temperature modulation.

### 2.4 Non-invasive cochlear-targeted temperature management

The custom cooling device used for these studies was repurposed from the original design used for localized cooling during cochlear implant surgeries (Tamames et al., 2016). The device modifications included a surface cooling attachment to non-invasively and bilaterally induce mild hypothermia (target temperature: 31–33°C) in the rat cochleae. The surface cooling attachment consisted of a 0.11” ID × 0.15” OD silicon collar. Local cooling was achieved by the regulating temperature of the circulating fluorocarbon through the collar with a custom thermoelectric controller (TE Technology Inc., TC720) controlled using a LabVIEW user interface.

TTM protocols were performed on anesthetized animals (Ketamine/Xylazine, 44/5 mg/kg) at 15 min post-noise exposure. Comparable doses of anesthetic cocktail solution were administered for the hypothermia (2.83 ± 0.24) and the normothermia (2 ± 0.24) groups (one-way ANOVA, *p* = 0.0668). Animals were placed on far infrared warming pads to regulate body temperature at 37°C (RightTemp Jr., Kent Scientific). A surface cooling attachment was placed around the rat’s neck and adjusted to fit over the ears without obstructing breathing. Following a hypothermic induction protocol (Schick et al., 2004), the hypothermia TTM protocol consisted of an initial 12-min induction period (fluorocarbon temperature decrease: 33°C to 5°C), followed by a 2-h continuous cooling period (fluorocarbon stable temperature: 5°C, inner ear or cochlear temperature maintained approximately 33°C), and finalized with a 12-min rewarming period (fluorocarbon temperature increase: 5°C to 33°C). In contrast, the normothermic TTM protocol consisted of a 2-h temperature maintenance period (fluorocarbon stable temperature: 33°C, inner ear or cochlear temperature maintained approximately 37°C). Following TTM protocols, animals were administered SR buprenorphine (1 mg/kg) to aid in pain management after noise exposure.

### 2.5 RNA extraction

Whole cochlear samples were collected from the noise-exposed animals, *Noise + Normothermia* and *Noise + Hypothermia* groups, at 24 h post-noise, and the age-matched *Unexposed Control* animals after euthanasia by CO_2_ inhalation, followed by decapitation (n = 3 rats/group). The cochleae were harvested in aseptic conditions using autoclaved tools cleaned with RNAlater solution. Only one cochlea per animal was used to reduce sample degradation time. The cochleae were then collected in RNA/DNA-free cryotubes, frozen in liquid nitrogen, and stored at −80°C prior to RNA extraction.

RNA was extracted from the cochlear samples using a Direct-zol RNA Microprep kit (Zymo Research, R2060). Cold TRIzol (1 mL, Invitrogen 15,596,026) was added to each cryotube to lyse the samples. Cochleae were homogenized for 20 s using Fisherbrand™ Model 150 Handheld Homogenizer equipped with a 7-mm stainless steel saw teeth bottom generator probe. Homogenized samples were centrifuged at 12,000 rpm for 12 min at 4°C to remove particulate debris. Supernatants were collected in RNA/DNA-free 1.5 mL tubes. An equal volume of ice-cold 100% EtOH was added to each tube. The mixtures were transferred into Zymo-Spin Columns and centrifuged for 30 s at 10.000. Next, 400 μL RNA Wash Buffer was added to the columns and centrifuged for 30 s. A mixture containing 5 μL of DNase and 75 μL of DNA digestion was added to each column and left at RT for 15 min. Columns were washed twice with 400 μL of Direct-zol RNA PreWash and once with 700 μL of RNA Wash Buffer. RNA was eluted with 20 μL of RNA/DNA-free water. RNA concentration was measured using NanoVue Plus Nanodrop (GE Healthcare Life Sciences). RNA concentrations between the different experimental groups averaged 41.49, 45.29, and 138.00 ng/μl for hypothermia-treated, normothermic noise-exposed, and unexposed controls (one-way ANOVA, *p* = 0.5018). RNA quality was further measured using the Bioanalyzer (Agilent Technologies), and an RNA integrity number (RIN) was determined based on the overall quantity of intact RNA.

### 2.6 Illumina sequencing

Next-generation RNA sequencing was used to analyze bulk cochlear transcriptome in the three groups at 24 h post-noise. RNA samples obtained from the three groups were obtained. Samples with a RIN > 6 proceeded to library preparation with the KAPA mRNA HyperPrep Kit (Roche). Libraries were sequenced on the Illumina NovaSeq 6000 in paired-end 150 bp reactions. Raw FASTQ files were aligned to the *Rattus norvegicus* genome (Rnor6.0) using the STAR algorithm (v2.5.2a) and genes quantified using the GeneCounts function within STAR against the annotated Ensembl v87 *Rattus norvegicus* gene build. Read-count matrices were read into R and processed using the “edgeR” package (v3.26.8). A multi-dimensional scaling plot was generated from calculated distances between samples based on the genes with the most heterogeneous expression. Negative binomial generalized linear models (GLMs) were fitted with estimated gene dispersions and differential expression determined using the GLM likelihood ratio test with a 5% false discovery rate (FDR) for differential expression testing.

### 2.7 Differential gene expression analysis

Differentially expressed genes (DEGs) were obtained between each pair of experimental groups to identify shared and unique genes, split by up- or downregulation. The union of DEGs among all group comparisons was clustered using the complete hierarchical clustering algorithm implemented in R. Differentially expressed gene transcripts between the three group were filtered by *p* < 0.05, counts per million (logCPM>0), and fold change (abs(FC) > 1.5) in the indicated comparisons. The Database of Annotation, Visualization and Integrated Discovery (DAVID v6.8;^1^ Sherman and Lempicki, 2009) was used to identify enriched gene ontology (GO) and KEGG pathway terms. Previously filtered downregulated or upregulated subset of DEGs from each group comparison was submitted into the DAVID functional annotation tool as a gene list with limited annotation by *Rattus norvegicus*. Obtained functional annotation charts were obtained for KEGG pathway analysis and GO terms for biological processes, cellular component, and molecular function. These records were screened to include a minimum count of four genes and an FDR < 0.05.

## 3 Results

### 3.1 Post-noise hypothermia treatment reduces ABR threshold shift

A comparison of the post-noise threshold shift from baseline was performed for the normothermic and hypothermic TTM groups (repeated measures two-way ANOVA, frequency*treatment, Figure 1A). Between-subject treatment and frequency effects were observed (*p* < 0.0001); however, the interaction effect was non-significant (*p* = 0.8133). Post-hoc contrasts revealed significant differences (*p* < 0.05) between the groups at all frequencies (Figure 1B). Overall, we observed similar and significant protection with hypothermia treatment against TTS and PTS causing noise exposure when compared to normothermic-treated group post-noise as in our companion study (Rincon Sabatino et al., 2023).

### 3.2 Identification of differentially expressed genes following acute noise exposure

**Figure 2:**
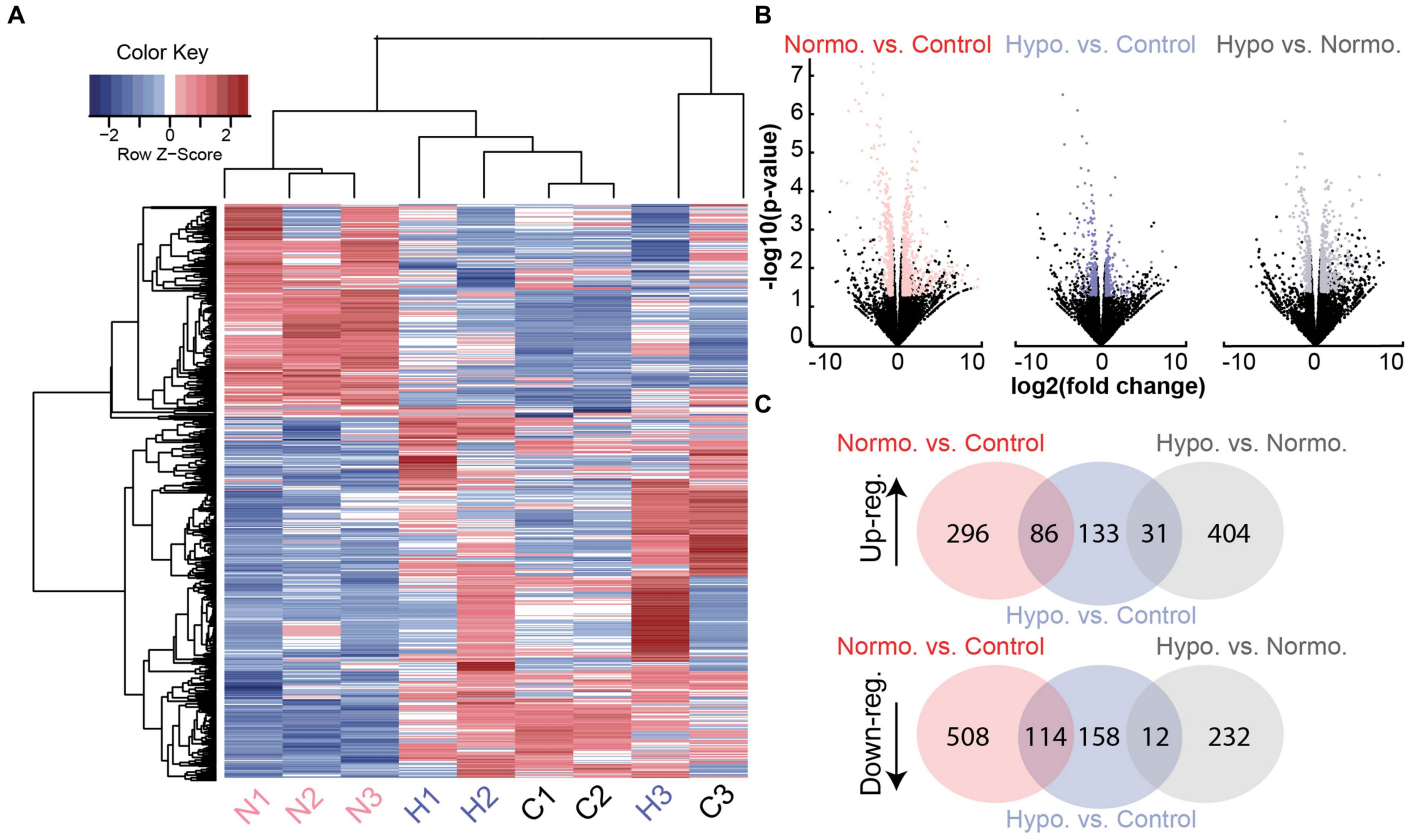
Differentially expressed gene transcripts from Noise + Hypothermia, Noise + Normothermia, and unexposed control groups. **(A)** Heatmap dendrogram of DEGs at 24 h in the three groups: normothermia (N1, N2, N3, pink), hypothermia (H1, H2, H3), and controls (C1, C2, C3). Hierarchical clustering of gene rows indicated expression patterns between the samples with indicated Z-scores of above-average (red) or below-average (blue) expression levels. **(B)** Gene counts from all group comparisons visualized by volcano plot indicating biological significance as a measure of nominal value of *p* and fold change. Out of 32,494 total genes compared between the groups, filtered protein-coding DEGs are highlighted per group comparison indicating a value of *p* < 0.05 and abs(log2 fold change) ≥ 1.5. Multiple testing corrections used the Benjamini and Hochberg method with the false discovery rate (FDR) calculated for each gene. Illustrated group comparisons are shown for normothermia versus control (NvC, red), hypothermia versus control (HvC, blue), and hypothermia versus normothermia (HvN, black). **(C)** Venn diagrams showing all shared and unique DEGs in the three group comparisons separated by upregulation (Up-reg.) and downregulation (Down-reg.).

We identified differentially expressed genes (DEGs) between each pair of experimental groups (hypothermia vs. normothermia, normothermia vs. control, hypothermia vs. control). A comparison of protein-coding genes between the groups revealed a total of 697 DEGs (statistical cutoff of *p* < 0.05 and logCPM>0 with a fold change of ±1.5, Figures 2A,B). Changes in gene expression that were similar for the normothermia and hypothermia groups were removed to focus analysis on mechanisms specific to hypothermic protection and identify possible hypothermia-induced cellular stress (Figure 2C).

### 3.3 Differential gene expression: hypothermia versus normothermia

Of the 404 uniquely upregulated genes between hypothermia versus normothermia (HvN) animals, the top 20 DEGs in terms of significance (FDR) for up- and downregulation are illustrated in Table 1. Three of these top upregulated DEGs are involved in regulating muscle contraction, namely, Tnnt2, Tnni2, and Atp2a1. Of the remaining top upregulated DEGs, two genes are members of the S100 calcium-binding family, S100a4 and S100b, which may be primarily expressed in Schwann cells and satellite glial cells and contribute to neuronal damage repair and regeneration (Locher et al., 2014; Lei and Tang, 2017). Among the top 20 downregulated DEGs are chemokines Cxcl11, Cxcl9, and Cxcl10. These cytokines are involved in many pathways, including cytokine–cytokine interactions, Toll-like receptor signaling, cellular metal ion homeostasis, humoral immune complement response, and chemokine signaling pathways.

**Table 1:** Hypothermia versus normothermia: top significant up- and down-regulated DEGs.

	Symbol	Name	logFC
1	*Mb*	Myoglobin	7.13
2	*Acta1*	Actin, alpha 1, skeletal muscle	4.90
3	*Kazald1*	Kazal-type serine peptidase inhibitor domain 1	1.25
4	*Ctxn3*	Cortexin 3	1.13
5	*Otos*	Otospiralin	1.75
6	*Slc41a3*	Solute carrier family 41, member 3	1.46
7	*S100b*	S100 calcium-binding protein B	1.40
8	*Aard*	Alanine and arginine rich domain containing protein	1.37
9	*Krt15*	Keratin 15	1.09
10	*Hhatl*	Hedgehog acyltransferase-like	1.43
11	*Tnni2*	Troponin I2, fast skeletal type, actin-binding activity	3.82
12	*Ckm*	Creatine kinase, M-type	5.10
13	*Npas2*	Neuronal PAS domain protein 2	1.60
14	*Mylpf*	Myosin light chain, phosphorylatable, fast skeletal muscle	4.19
15	*S100a4*	S100 calcium-binding protein A4	1.14
16	*Atp2a1*	ATPase sarcoplasmic/endoplasmic reticulum Ca^2+^ transporting 1	4.09
17	*Tnnt2*	Troponin T2, cardiac type	0.94
18	*Hs3st6*	Heparan sulfate-glucosamine 3-sulfotransferase 6	1.20
19	*Mfap5*	Microfibril-associated protein 5	1.04
20	*Tmem72*	Transmembrane protein 72	1.91
1	*Cxcl11*	C-X-C motif chemokine ligand 11	−3.42
2	*Lcn2*	lipocalin 2	−2.88
3	*Timp1*	TIMP metallopeptidase inhibitor 1	−1.48
4	*Cxcl9*	C-X-C motif chemokine ligand 9	−2.03
5	*Kng2*	Kininogen 2	−1.21
6	*Vmo1*	Vitelline membrane outer layer 1 homolog	−2.11
7	*Ecel1*	Endothelin-converting enzyme-like 1	−3.26
8	*Ifit3*	Interferon-induced protein with tetratricopeptide repeats 3	−1.58
9	*Chi3l1*	Chitinase 3 like-1	−1.28
10	*C4b*	Complement C4B	−1.64
11	*Prg4*	Proteoglycan 4	−1.87
12	*Il17rb*	Interleukin 17 receptor B	−1.61
13	*LOC299282*	Serine protease inhibitor	−0.86
14	*Klhl40*	Kelch-like family member 40	−1.39
15	*Osmr*	Oncostatin M receptor	−1.31
16	*Mx2*	MX dynamin-like GTPase 2	−1.08
17	*Cxcl10*	C-X-C motif chemokine ligand 10	−2.21
18	*Hpx*	Hemopexin	−1.37
19	*Csf2rb*	Colony stimulating factor 2 receptor subunit beta	−0.93
20	*C9*	Complement C9	−1.53

Summary of top up- (red) and down- (blue) regulated genes involved in hypothermic neuroprotection of noise-induced hearing loss. Hypo. versus Normo. DEGs. logFC, log fold change.

**Figure 3:**
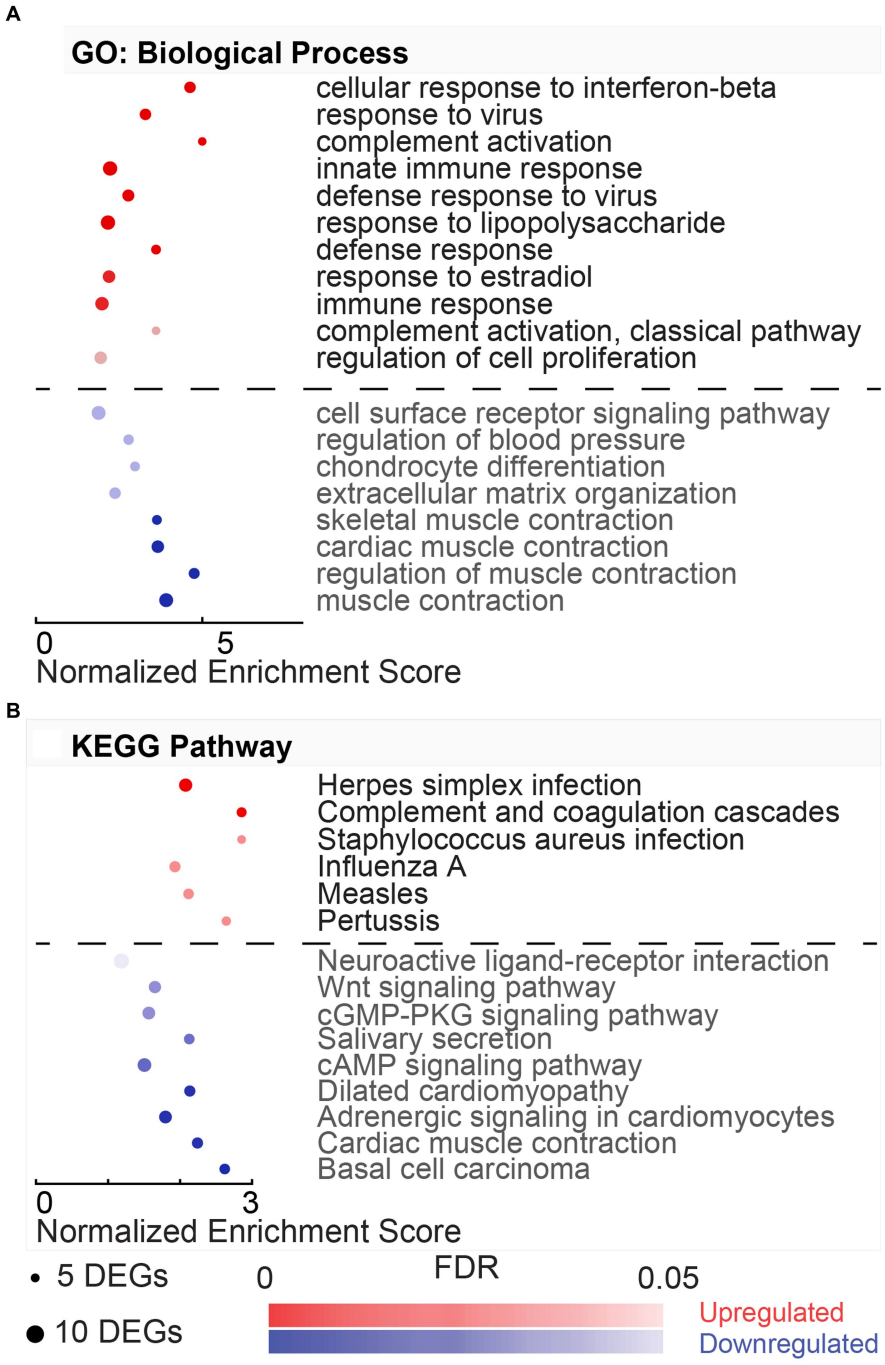
Gene ontology and pathway enrichment associated with hypothermic neuroprotection post-noise. **(A)** DAVID gene ontology (GO) biological process enrichment analysis for upregulated (red) and downregulated (blue) DEGs in the normothermia versus hypothermia comparison. Enriched biological processes are listed on the y-axis, and normalized enrichment scores associated with the process are listed on the x-axis. Dot size indicates the number of genes included in each category, and dot color indicates significance of the enrichment (FDR color bar, adjusted value of *p* < 0.05). **(B)** Dot plot with resultant enriched KEGG pathways from DAVID analysis from the same subset of genes dysregulated with post-noise cochlear cooling (upregulation, red, downregulation, blue).

The GO terms for the biological process include three upregulated and fifteen downregulated processes (Figure 3A). In the biological processes associated with the upregulated DEGs, the top (FDR = 9.53E-04) process was the regulation of muscle contraction (GO:0006937, 1.99%), including DEGs, such as Atp2a1, Mylk2, Tnnc2, Tnni2, Tnnt1, Tnnt2, and Tnnt3. Most downregulated processes were involved in the immune response and included key regulators, including transcription factors, Stat1 and Stat2, and chemokines, Cxcl9, Cxcl10, and Cxcl11. The top downregulated biological process (FDR = 1.05E-08) was cellular response to interferon-beta (GO:0035458, 4.70%). This process involved DEGs, such as Ifi47, Ifit3, Igtp, Irgm, Gbp1, Gbp2, Gbp4, Hcn1, and Stat1.

The KEGG pathways associated with the HvN are included in Figure 3B. These resultant pathways from the upregulated DEGs include oxidative phosphorylation (rno00190, 4.77%), Parkinson’s disease (rno05012, 3.98%), Alzheimer’s disease (rno05010, 3.98%), non-alcoholic fatty liver disease (NAFLD) (rno04932, 3.45%), Huntington’s disease (rno05016, 3.71%), metabolic pathways (rno01100, 11.14%), and cardiac muscle contraction (rno04260, 2.12%). The oxidative phosphorylation pathway (rno00190, 4.77%) was highly enriched in this comparison (FDR = 2.18E-07). This pathway was associated with 18 upregulated DEGs, including several cytochrome c oxidase units (Cox7a2l2, Cox7b, Cox7c, Cox8b), ubiquinone-related genes (Ndufa6, Ndufa4, Ndufa2, Ndufc1, Ndufs5, Uqcrb, Uqcr10, Uqcr11), and ATP synthase-related genes (Atp5e, Atp5i, Atp5j, Atp5j2, Atp5l). The KEGG pathways associated with the downregulated DEGs include Herpes simplex infection (rno05168, 5.98%), influenza A (rno05164, 4.70%), Toll-like receptor signaling pathway (rno04620, 3.42%), hepatitis C (rno05160, 3.42%), RIG-I-like receptor signaling pathway (rno04622, 2.56%), and measles (rno05162, 3.42%).

In this hypothermia versus normothermia comparison, upregulated DEGs included cellular component terms, such as extracellular space, extracellular exosome, mitochondrial proton-transporting ATP synthase complex, troponin complex, axon, mitochondrial inner membrane, proteinaceous extracellular matrix, extracellular region, and extracellular matrix (Supplementary Table S1). The mitochondrial proton-transporting ATP synthase complex (GO:0005753, 1.86%) and mitochondrial inner membrane (GO:0005743, 4.51%) included several ATP synthase-related genes (Atp5e, Atp5j, Atp5j2, Atp5l) and other members of the mitochondrial respiratory chain complex (Cox7c, Uqcrb, Uqcc2, Uqcr10, Uqcr11), whereas the downregulated DEGs were enriched for GO cellular component terms, such as extracellular space, neuron projection, and neuronal cell body (Supplementary Table S1). Only two terms were enriched for molecular function in the comparison of down- and upregulated hypothermia versus normothermia. For the upregulated DEGs, RAGE receptor binding (GO:0050786, 1.33%) was the only resultant molecular function (FDR = 0.0083), and for the downregulated DEGs, only GTPase activity (GO:0003924, 5.13%) was significant (FDR = 0.0117).

### 3.4 Possible cellular targets for hypothermic treatment of NIHL

Of these previously specified DEGs pertaining to hypothermia versus normothermia biological processes, a single-cell target was estimated using the gEAR portal to observe enhanced expression in a noise-exposed mouse model from Milon et al. (2021). Only the dysregulated DEGs observed in both HvN and the mouse scRNA-seq dataset are included in Table 2. This table shows the up- and down-regulated DEGs after PTS-inducing noise in a subset of cells found in the organ of Corti of male CBA/CaJ mice. The upregulated DEGs associated with hypothermia versus normothermia enriched biological processes include Car2, Cox8b, Crip1, Slc4a10, Tnnc2, Tnnt1, Tnnt2, Uqcr11, and Uqcrb. For the top dysregulated DEG in this comparison, Car2, an increase in expression after noise exposure, was observed only in OHCs, with observed downregulation in SGNs, LW cells, monocytes, neutrophils, and beta cells. Cox8b showed noise-induced downregulation in SGNs, LW cells, and inflammatory cells. After noise exposure, the fibrocyte-specific Slc4a10 showed a decreased expression in the lateral wall (LW). Previously discussed troponins involved in functions of calcium binding and muscle contraction (Tnnc2, Tnnt1, Tnnt2) showed noise-induced dysregulation in OHCs, SCs, LW cells, monocytes, and neutrophils. Noise-induced decrease was observed in OHCs for Tnnc2 and Tnnt1 and in SCs for Tnnt1 and Tnnt2. Electron transport-associated DEGs Uqcr11 and Uqcrb showed mixed expression in B cells, SGNs, and LW cell types but distinct upregulation in inflammatory monocytes and neutrophils.

**Table 2:** Single-cell targets from differentially expressed genes obtained from gene ontology biological processes for Noise + Hypothermia versus Noise + Normothermia.

Name		logFC	Value of *p*	Function	OHCs	SCs	SGNs	LW	Mono	Nphil	B-cell
1	*Car2*	1.07	1.2E-03	Zinc binding	2		−1	−1	−1	−1	−1
2	*Cox8b*	3.31	2.5E-03	Electron transport			−1	−1	−1	−1	−1
3	*Crip1*	0.67	3.7E-03	Zinc binding			2	2	2	2	2
4	*Slc4a10*	0.86	9.2E-03	Ion transport (Na)			2	−1			
5	*Tnnc2*	2.72	7.1E-04	Calcium binding	−1						
6	*Tnnt1*	2.62	1.6E-03	Muscle contraction	−1	−1		2	2	2	
7	*Tnnt2*	0.94	5.1E-04		2	−1		2			
8	*Uqcr11*	0.59	7.3E-03	Electron transport			1	1	2	2	−1
9	*Uqcrb*	0.63	9.7E-03				1	1	2	2	2
1	*Cdo1*	−0.93	1.4E-03	Iron binding			1	1			
2	*Chst11*	−0.67	6.0E-03	Carbohydrate metabolism			−1	1	2		
3	*Csf2rb*	−0.93	5.3E-04	Cytokine activity	2				2	2	
4	*Ccxl10*	−2.21	4.1E-04		2	2			2		
5	*Fosl1*	−1.96	1.3E-03	DNA binding				2			
6	*Gbp2*	−1.23	5.0E-03	GTP binding				2			
7	*Gbp4*	−1.62	4.4E-03					2			−1
8	*Hcn1*	−1.01	9.1E-03	Ion transport (Na, K)			2				
9	*Hmgcs2*	−0.94	2.2E-03	Cholesterol metabolism		2	2	2			
10	*Ifi47*	−1.19	2.3E-03	GTP binding							2
11	*Ifit3*	−1.58	1.5E-04	RNA binding			−1	2	2	2	2
12	*Igtp*	−0.99	1.5E-03	GTP binding			−1	1			−1
13	*Il18bp*	−1.04	1.8E-03	Cytokine activity				2			
14	*Isg15*	−1.25	8.1E-04	Integrin-mediated signaling			1	2	2		−1
15	*Kng2*	−1.21	4.8E-05	Vasodilation		−1					
16	*Lbp*	−1.54	9.2E-04	Lipid transport			2	2		−1	
17	*Lcn2*	−2.88	2.2E-05	Ion transport (Fe)			2	2		−1	
18	*Mx2*	−1.08	3.6E-04	GTP binding	2	2					
19	*Oasl2*	−0.68	8.6E-03	RNA binding				2	2		
20	*Osmr*	−1.31	3.1E-04	Cytokine activity	2	2		1			
21	*Pappa*	−1.50	7.6E-03	Zinc binding	−1		1				
22	*Ptgs2*	−1.21	9.1E-03	Iron binding	2	2					
23	*Serping1*	−0.86	7.0E-03	Complement binding			2	2			
24	*Slc9a3*	−0.75	9.7E-03	Ion transport (Na)	−1	2					
25	*Slpi*	−1.07	8.6E-03	DNA binding	2	−1				2	−1
26	*Socs3*	−1.24	7.1E-03	Growth regulation	2	2	1	1	−1		2
27	*Stat1*	−0.83	6.7E-04	DNA binding			−1	2	−1	−1	2
28	*Stat2*	−0.65	4.6E-03	DNA binding	2	2	−1	2			
29	*Tap1*	−0.94	3.1E-03	ATP binding				2	−1		
30	*Timp1*	−1.48	2.3E-05	Cytokine activity			2	2			
31	*Vgf*	−1.88	1.7E-03	Growth factor activity		−1	2				

Summary of cellular targets associated with dysregulated DEGs involved in hypothermic neuroprotection post-noise. The organ of Corti single-cell types includes outer hair cells (OHCs), supporting cells (SCs), spiral ganglion neurons (SGNs), lateral wall cell types (LW), and inflammatory cells, such as monocytes (Mono), neutrophils (Nphil), and beta cells (B-cell). The LW cell types include cells from the stria vascularis (marginal, intermediate, and basal cells) and the spiral ligament (perivascular endothelial cells, root cells, and fibrocytes), whereas the spiral ganglion neurons comprise Type 1 (1A, 1B, and 1C) and Type 2 SGNs. Upregulated expression after noise is indicated in red, and downregulated expression is indicated in blue. Mixed differential gene expression within the LW and SGN subtypes is indicated in gray. Hypo. versus Normo. DEGs. logFC, log fold change.

Of the DEGs downregulated with HvN, multiple cytokine-related DEGs, such as Cxcl10, Il18bp, Osmr, Lcn2, and Timp1, showed noise-induced upregulation in distinct cell types. Cxcl10 showed increased expression in OHCs, SCs, and monocytes, while Il18bp increase was limited to the LW. Lcn2 and Timp1 showed increased expression in both SGNs and LW cell types, but Lcn2 also had a noise-induced decrease in neutrophils. Osmr showed increased activity in OHCs and SCs with mixed expression in LW subtypes. Mixed expression was observed throughout cell types with DNA-binding cytokine regulators, Socs3, Stat1, and Stat2. A general increase was observed in OHCs, SCs, LW cells, and beta cells, and a decrease was observed in SGNs, monocytes, and neutrophils.

### 3.5 Differential gene expression: normothermia versus control

Of the 296 uniquely upregulated and the 508 uniquely downregulated genes in normothermic treatment groups compared to unexposed control (normothermia vs. control, NvC), the top 20 up- and downregulated genes by significance (value of *p*) are indicated in Table 3. Top genes upregulated with normothermia are related to immune and inflammatory pathways, including Ifit3, Isg15, Enpp3, Serping1, C4b, Stat1, and Parp14. Of the downregulated transcripts for this comparison, most DEGs were related to the cellular response to organic compounds, such as Aldh3a1, Car2, Pde2a, and Rapgef3. Additionally, two transcripts related to negative regulation of cell differentiation, Wnt3 and S100b, were also observed in the top 10 differentially downregulated genes. The fibrocyte-specific otospiralin (Otos, logFC = −1.81) also showed a significant downregulation compared to unexposed control samples.

**Table 3:** Normothermia versus control: top significant up- and down-regulated DEGs.

	Symbol	Name	logFC
1	*C4b*	Complement C4B	2.07
2	*Klhl40*	Kelch-like family member 40	1.80
3	*Isg15*	ISG15 ubiquitin-like modifier	1.88
4	*Serping1*	Serpin family G member 1	1.33
5	*Pou3f4*	POU class 3 homeobox 4	2.32
6	*Rasal2*	RAS protein activator like 2	6.80
7	*Clca4l*	Chloride channel calcium activated 4-like	6.10
8	*Ifit3*	Interferon-induced protein with tetratricopeptide repeats 3	2.22
9	*Csf2rb*	Colony stimulating factor 2 receptor subunit beta	1.41
10	*Aebp1*	Adipocyte enhancer-binding protein 1	1.26
11	*Serpine2*	Serpin family E member 2	1.19
12	*Scnn1a*	Sodium channel epithelial 1 subunit alpha	1.50
13	*Enpp3*	Ectonucleotide pyrophosphatase/phosphodiesterase 3	1.42
14	*Rsl1*	Regulator of sex limited protein 1	0.94
15	*Stat1*	Signal transducer and activator of transcription 1	0.87
16	*Phc3*	Polyhomeotic homolog 3	1.15
17	*Gbp1*	Guanylate-binding protein-1-encoding gene	1.85
18	*Parp14*	Poly (ADP-ribose) polymerase family, member 14	1.13
19	*Mx2*	MX dynamin-like GTPase 2	1.38
20	*Hmgcs2*	3-Hydroxy-3-methylglutaryl-CoA synthase 2	1.12
1	*Kazald1*	Kazal-type serine peptidase inhibitor domain 1	−1.61
2	*Sfrp5*	Secreted frizzled-related protein 5	−2.52
3	*Aard*	Alanine and arginine rich domain containing protein	−1.93
4	*S100b*	S100 calcium-binding protein B	−1.74
5	*Slc41a3*	Solute carrier family 41, member 3	−1.96
6	*Rflnb*	Refilin B	−1.19
7	*Wnt3*	Wnt family member 3	−3.28
8	*Sbspon*	Somatomedin B and thrombospondin, type 1 domain containing	−1.55
9	*Car2*	Carbonic anhydrase 2	−1.18
10	*Aldh3a1*	Aldehyde dehydrogenase 3 family, member A1	−0.96
11	*Otos*	Otospiralin	−1.81
12	*Rapgef3*	Rap guanine nucleotide exchange factor 3	−1.34
13	*LOC102550391*	Glutathione S-transferase alpha-3-like	−1.28
14	*Pde2a*	Phosphodiesterase 2A	−1.12
15	*Cnmd*	Chondromodulin	−1.07
16	*Ippk*	Inositol-pentakisphosphate 2-kinase	−1.20
17	PDE4C	Phosphodiesterase 4C	−1.12
18	HS3ST6	Heparan sulfate-glucosamine 3-sulfotransferase 6	−1.45
19	TP53I11	Tumor protein p53 inducible protein 11	−0.90
20	SLC6A13	Solute carrier family 6 member 13	−1.38

Summary of top up-(red) and down-(blue) regulated genes involved in acute noise response with post-noise normothermia TTM as compared to unexposed control animals. Normo. versus control DEGs. logFC, log fold change.

**Figure 4:**
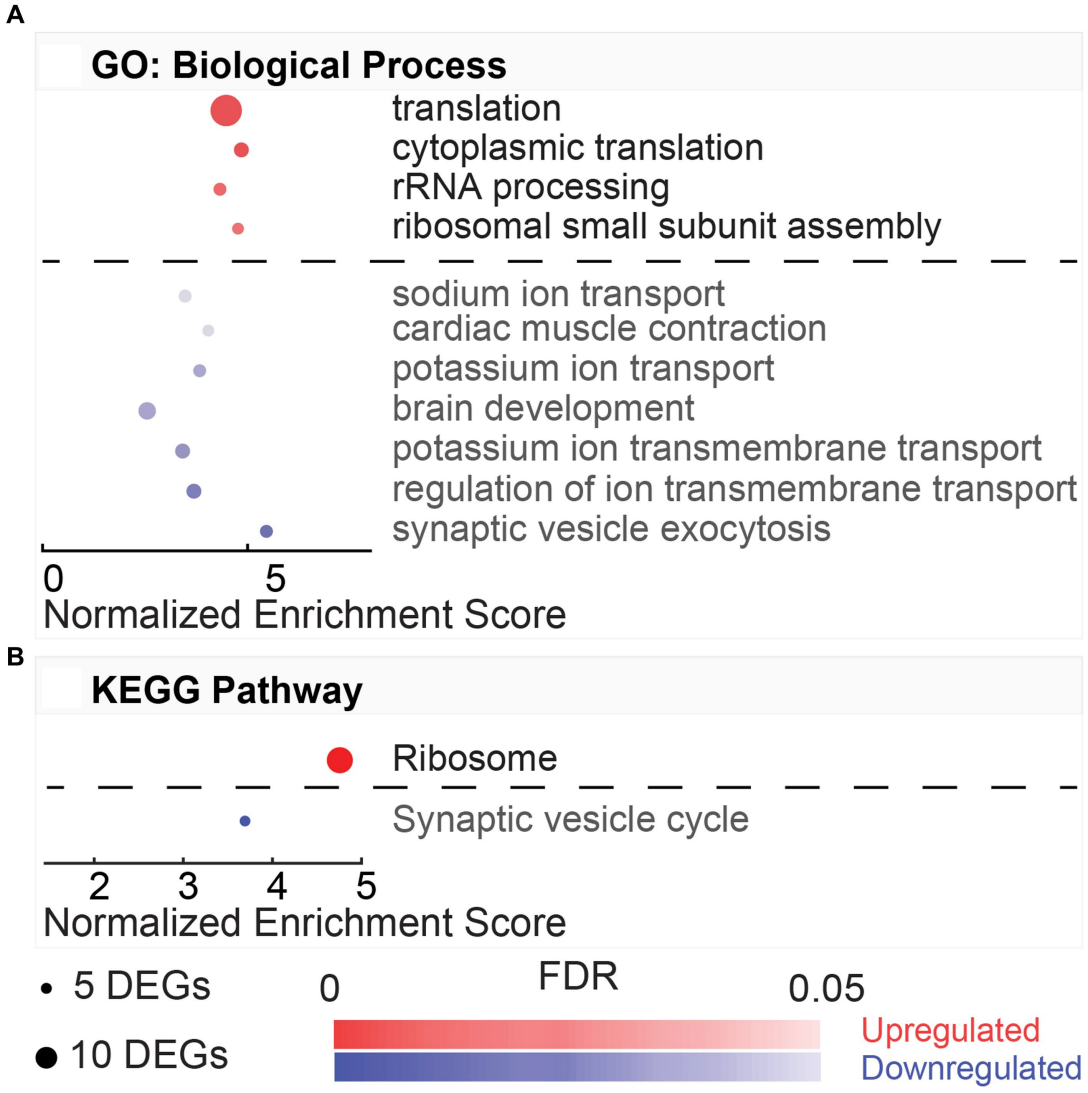
Gene ontology and pathway enrichment with post-noise normothermia TTM. **(A)** Enriched gene ontology (GO) biological processes associated with upregulated (red) and downregulated (blue) DEGs in the normothermia versus unexposed control comparison. **(B)** Enriched KEGG pathways associated at 24 h post-exposure are listed on y-axis. The x-axis indicates normalized enrichment score acquired from the DAVID functional annotation tool. False discovery rate (FDR) color bar indicates adjusted value of *p* of upregulated (red) and downregulated (blue) categories with lighter colors suggesting less significance. The size of the dots denotes the number of relevant DEGs included in the pathway.

Gene ontology analysis offered by DAVID captured eleven upregulated and eight downregulated biological processes with a minimum gene set of 5 DEGs and an FDR < 0.05 (Figure 4A). Of the eleven upregulated processes identified by DAVID gene ontology for this NvC comparison, most terms were involved in immunological and inflammatory functions (Figure 4A). Several of these processes are modulated by cytoplasmic transcription factors and vital mediators of interferon signaling, Stat1, Stat2, and Stat3. The top upregulated biological process, cellular response to interferon-beta (GO:0035458, 3.72%), included regulators, such as Ifit3, Ifi47, and Igtp. DAVID ontology identified two complement activation pathways, including GO:0006956 (2.03%) and GO:0006958 (2.03%), including several DEGs in the classical pathway, such as C1s, C1r, C2, C4a, C4b, and C6. However, the alternative pathway associated with complement component factor b was also upregulated in the normothermia animals. The top four downregulated pathways involved systems of muscle contraction (GO:0006936, 3.19%), including several actin-myosin regulators that are activated in response to increased intracellular Ca^2+^ (Figure 4A). These include a subset of the genes encoding skeletal-type troponin-Ts, such as Tnnt2, Tnnt3, Tnni1, Tnni2, and Tnnc2, and genes encoding myosin light chains, such as Myl1, Myl2, and Myl3. KEGG pathway analysis performed with DAVID identified six upregulated and nine downregulated pathways for the NvC comparison (Figure 4B).

We then looked at the cellular localization of the upregulated DEGs in the normothermia-treated group. Gene ontology results indicated enriched expression of six cellular components, namely, the apical plasma membrane, membrane, neuronal cell body, extracellular space, apical part of cell, and extracellular region (Supplementary Table S2). Enriched expression in the cellular membrane and extracellular space was consistent with upregulated DEGs being mainly associated with immune defense and inflammatory signaling. Enriched terms from the cellular component gene ontology analysis included 22 GO terms from the downregulated DEGs alone (Supplementary Table S2). By order of decreasing significance, these terms include extracellular space, proteinaceous extracellular matrix, extracellular region, Z disk, extracellular exosome, troponin complex, myofibril, basement membrane, extrinsic component of external side of plasma membrane, anchored component of membrane, integral component of plasma membrane, cell surface, I band, extracellular matrix, myosin complex, sarcomere, sarcoplasmic reticulum, contractile fiber, M band, axon, lysosome, and collagen trimer. Most DEGs were associated with extracellular localization, including 97 DEGs in extracellular space, 48 DEGs associated with the extracellular region, and 107 associated with extracellular exosome, with only 11 DEGs repeated between the distinct cellular components. Another cellular location associated with the downregulated DEGs included the troponin complex and myofibril, seen expressly in the highly dysregulated troponins and myosin light and heavy chain DEGs. As for the molecular functions that these DEGs carry out, GTPase activity was the only term associated with the list of upregulated DEGs (Supplementary Table S2). The enriched terms from the set of downregulated DEGs included calcium ion binding, structural constituent of muscle, actin binding, frizzled binding, heparin binding, and titin binding (Supplementary Table S2). The DEGs pertaining to this category include calcium- and actin-binding troponins (Tnnc2, Tnnt2, Tnnt3, Tnni1, Tnni2) and myosins (Myl1, Myl2, Myl3, Myo5c, Myh7).

### 3.6 Differential gene expression: hypothermia versus control

Of the 133 uniquely upregulated and the 158 uniquely downregulated genes within the hypothermia-treated compared to unexposed control (hypothermia vs. control, HvC) conditions, the top 20 DEGs in terms of highest significance are illustrated in Table 4. Upregulated DEGS included inflammatory mediators Rarres2, Nfkbiz, and Sele. While the top upregulated DEGs, Cdhr3 and Dnah6, are cilia-associated genes identified in middle ear pathology (Tian et al., 2017; Mulay et al., 2021), they are not associated with any known inner ear pathology. Of the downregulated DEGs in the same comparison group, two top DEGs, Lypd1 and Lypd6, are involved in the negative regulation of receptor activity. Several downregulated DEGs are also involved in signaling and differentiation, including Sox8, Sgpp2, and Ky.

**Table 4:** Hypothermia versus control: top significant up- and down-regulated DEGs.

	Symbol	Name	logFC
1	*Cdhr3*	Cadherin-related family member 3	3.72
2	*Dnah6*	Dynein, axonemal, heavy chain 6	3.12
3	*LOC687679*	Similar to small nuclear ribonucleoprotein polypeptide G	1.15
4	*Sele*	Selectin E	1.28
5	*Sec61b*	SEC61 translocon subunit beta	0.72
6	*Rarres2*	Retinoic acid receptor responder 2	0.60
7	*LOC103694169*	60S ribosomal protein L39	1.66
8	*Ostc*	Oligosaccharyltransferase complex non-catalytic subunit	0.65
9	*Nfkbiz*	NFKB inhibitor zeta	0.87
10	*Agr2*	Anterior gradient 2	2.64
11	*Bst2*	Bone marrow stromal cell antigen 2	0.99
12	*Rassf6*	Ras association domain family member 6	0.91
13	*AABR07026997.1*		1.00
14	*Bola2*	Bola family member 2	0.69
15	*Txn1*	Thioredoxin 1	0.86
16	*Tceal9*	Transcription elongation factor A like 9	0.60
17	*AABR07035791.1*		2.94
18	*Sat1*	Spermidine/spermine N1-acetyl transferase 1	0.71
19	*Prrg1*	Proline-rich and Gla domain 1	0.76
20	*Nbeal1*	Neurobeachin-like 1	1.35
1	*Kcns1*	Kv channel, modifier subfamily S, member 1	−1.15
2	*Cntnap4*	Contactin-associated protein family member 4	−1.01
3	*Fam78a*	Family with sequence similarity 78, member A	−0.92
4	*Esrrg*	Estrogen-related receptor gamma	−0.76
5	*Zfp804a*	Zinc finger protein 804A	−0.82
6	*Rps6ka2*	Ribosomal protein S6 kinase A2	−0.60
7	*Lypd1*	Ly6/Plaur domain containing 1	−0.81
8	*Cmya5*	Cardiomyopathy associated 5	−1.60
9	*Ky*	Kyphoscoliosis peptidase	−1.65
10	*Fbxo40*	F-box protein 40	−1.43
11	*Sv2a*	Synaptic vesicle glycoprotein 2a	−1.10
12	*Ina*	Internexin neuronal intermediate filament protein, alpha	−1.02
13	*Sox8*	SRY-box transcription factor 8	−0.77
14	*Cracd*	Capping protein inhibiting regulator of actin dynamics	−0.63
15	*Sgpp2*	Sphingosine-1-phosphate phosphatase 2	−0.74
16	*Lypd6*	Ly6/Plaur domain containing 6	−0.77
17	*Thbs1*	Thrombospondin 1	−0.86
18	*Abcg4*	ATP binding cassette subfamily G member 4	−0.83
19	*Map3k13*	Mitogen-activated protein kinase kinase kinase 13	−0.67
20	*Rcan2*	Regulator of calcineurin 2	−0.60

Summary of top up- (red) and down-(blue) regulated genes observed with post-noise cochlear hypothermia induction as compared to unexposed control animals. Hypo. versus control DEGs. logFC, log fold change.

**Figure 5:**
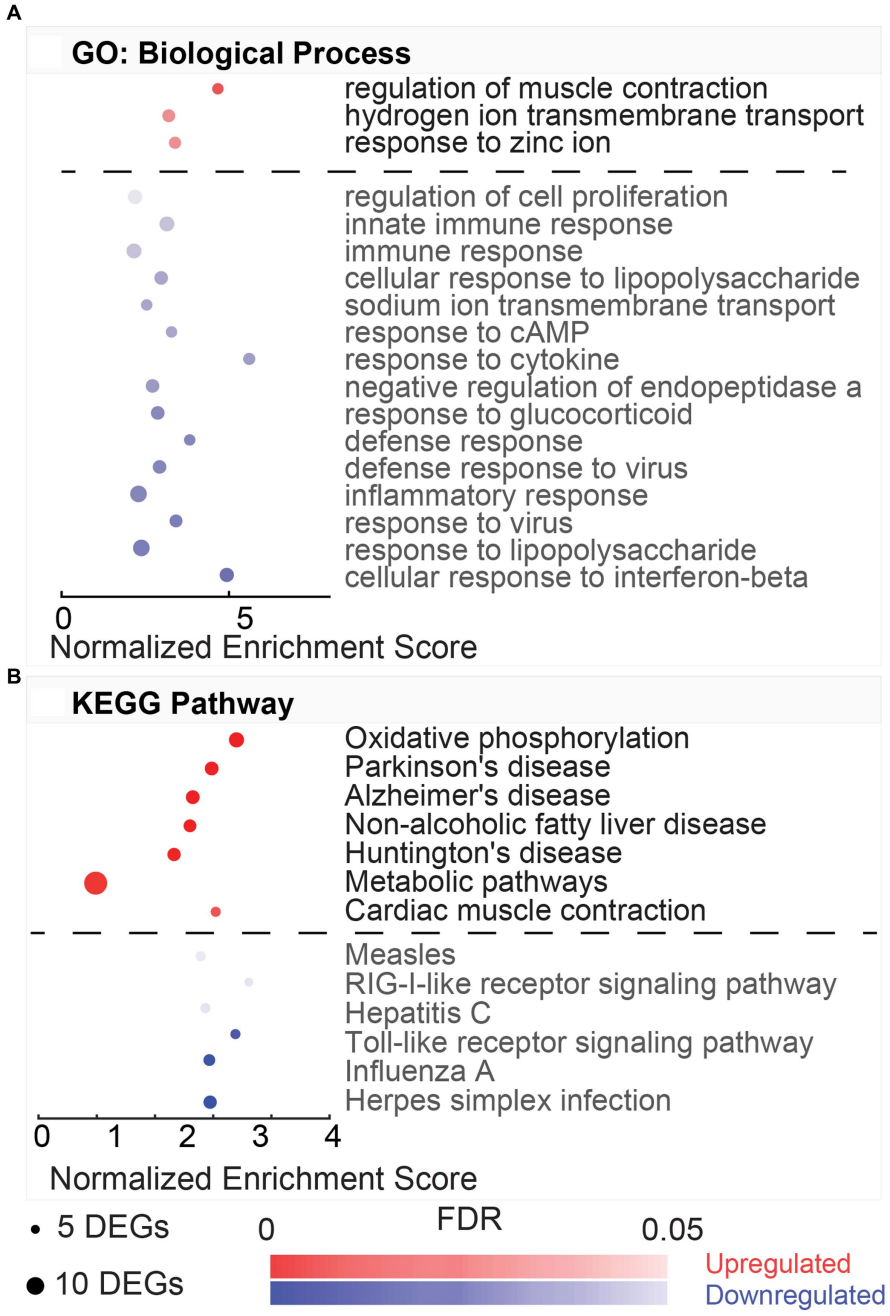
Gene ontology and pathway enrichment with post-noise hypothermia TTM. **(A)** Dot plot indicating results of DAVID gene ontology (GO) biological process enrichment analysis. DEGs obtained at 24-h post-noise were compared between animals receiving hypothermia and unexposed control animals. Analysis was performed for separate upregulated (red) and downregulated (blue) DEGs in this comparison. Biological processes associated with up- or downregulation are listed on the y-axis with x-axis indicating the normalized enrichment. Size of the dots indicates the number of DEGs included in each biological process, and the color indicates the significance of the process enrichment indicated the FDR-adjusted value of *p* (color bar). **(B)** Dot plot for the enriched KEGG pathways from the DAVID enrichment analysis for the hypothermia versus control comparison indicating the upregulated (red) and downregulated (blue) pathways.

DAVID gene ontology analysis performed on DEGs from HvC comparisons identified four upregulated and seven downregulated biological processes following the same criteria from previous comparisons (5 DEG minimum, FDR < 0.05,) illustrated in Figure 5A. Upregulated pathways included translation (GO:0006412, 32.43%), cytoplasmic translation (GO:0002181, 7.21%), rRNA processing (GO:0006364, 5.41%), and ribosomal small subunit assembly (GO:0000028, 4.50%). DEGs involved in these processes include several ribosomal protein-coding genes, such as Rpl9, Rpl11, Rpl26, Rpl36, Rps16, Rps17, Rps24, Rps27l, and Rps28. In this comparison, synaptic vesicle exocytosis was the top downregulated (FDR = 4.67E-04) process (GO:0016079, 3.59%). This process included the following subset of DEGs: Apba2, Cadps, Cplx1, Rab3a, Syt2, and Trim9. Other downregulated processes involved transmembrane ion transport, such as regulation of ion transmembrane transport (GO:0034765, 4.79%), potassium ion transmembrane transport (GO:0071805, 4.79%), potassium ion transport (GO:0006813, 3.59%), and sodium ion transport (GO:0006814, 3.59%). DEGs involved in these biological processes were linked to hyperpolarization-activated sodium and potassium channels (Hcn4, Hcn2), potassium voltage-gated channels (Kcna2, Kcnc1, Kcnc3, Kcnh2, Kcns1), and ATP-dependent sodium exchange (Atp1a3, Atp1b1). Only two KEGG pathways were associated with the comparison (Figure 5B). The only KEGG pathway associated with the upregulated DEGs in this comparison was ribosome (rno03010, 32.43%). This highly dysregulated pathway (FDR = 3.02E-42) included a subset of 36 genes, including Rpl9, Rpl36, Rps3, Rps11, Rps12, Rps15a, and Rps20.

GO terms for cellular component were also compared between HvC control animals (Supplementary Table S3). Cellular component terms associated with the upregulated DEGs include the cytosolic large ribosomal subunit, cytosolic small ribosomal subunit, ribosome, small ribosomal subunit, focal adhesion, membrane, and extracellular exosome. Most DEGs in the upregulated comparison include previously mentioned ribosome-associated genes from the ribosome KEGG pathway and ribosomal and translation-associated biological processes. For the cellular component terms associated with downregulation, many DEGs were present in the synaptic vesicle, terminal bouton, myelin sheath, cell junction, axon, synapse, axon terminus, dendrite, neuronal cell body, synaptic vesicle membrane, postsynaptic density, presynaptic membrane, voltage-gated potassium channel complex, intercalated disk, perikaryon, and cytoskeleton. Both the top downregulated pathway and most downregulated DEGs are related to synaptic complex. The comparison of molecular function terms resulted in three enriched terms for the upregulated DEGs and four enriched terms for the downregulated DEGs (Supplementary Table S3). The molecular function terms associated with the upregulated DEGs include structural constituent of ribosome, poly(A) RNA binding, and RNA binding. The DEGs associated with potassium channel activity include previously mentioned genes Hcn2, Hcn4, Kcna2, Kcnc1, Kcnc3, Kcnh2, Kcnj11, and Kcns1. In contrast, the terms associated with downregulation included protein binding, voltage-gated potassium channel activity, protein kinase binding, and delayed rectifier potassium channel activity.

### 3.7 Differential gene expression: comparison of all groups

**Figure 6:**
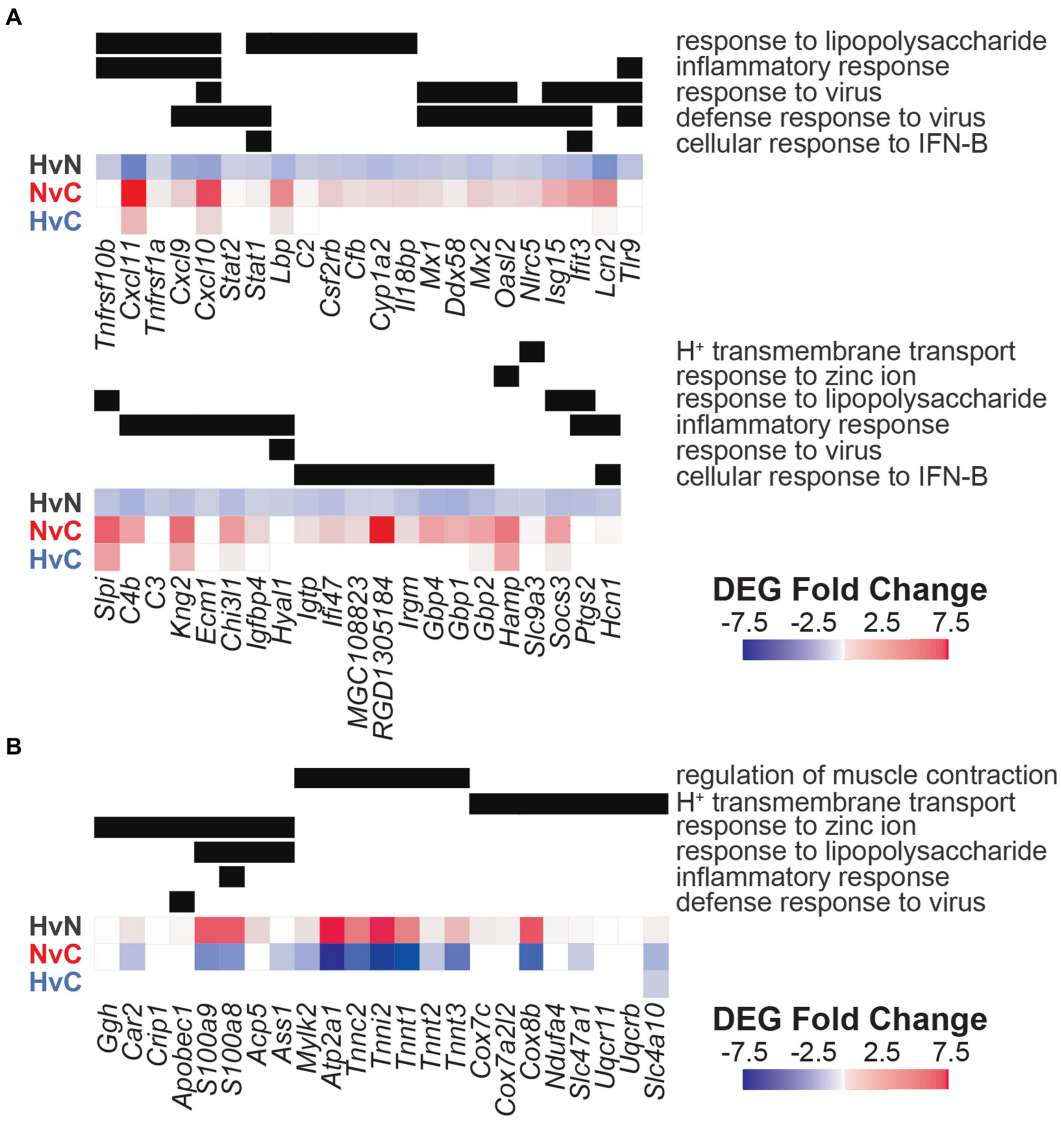
Differentially expressed genes from corresponding gene ontology analysis in hypothermic neuroprotection post-noise. Enriched biological processes associated with DEGs downregulated **(A)** or upregulated **(B)** with post-noise cochlear cooling are presented on the y-axis (right). Fold change observed for specific genes within these pathways is portrayed with a heatmap (red = upregulated, blue = downregulated) for the hypothermia versus normothermia (HvN, black), normothermia versus control (NvC, red), and hypothermia versus control (HvC, blue) comparisons. DEG inclusion in relevant biological pathways is indicated with a black bar. The corresponding FC values are further highlighted in Supplementary Table S4.

The dysregulated DEGs associated with the enriched hypothermia versus normothermia (HvN) biological processes are listed in Figure 6, along with the calculated fold change across the other comparisons of hypothermia versus control (HvC, Figure 6A) and normothermia versus control (NvC, Figure 6B). Many DEGs have overlapping functions in distinct biological processes, such as the cytokine Cxcl10, which is involved in several top inflammatory processes. Other highly overlapping inflammatory-related DEGs include Cxcl9, Stat1, Ifit3, and Tlr9. For theses DEGs, an upregulation in the NvC comparison was mirrored by a downregulation in the HvN comparison. However, an upregulation in the HvC comparison was still observed for Cxcl10 and Tlr9. In the upregulated HvN comparison, there were no observable overlapping DEGs for regulation of muscle contraction, hydrogen ion transmembrane transport, and response to zinc ion. The corresponding FC values for each comparison and each gene are detailed in Supplementary Table S4.

### 3.8 Hypothermic protection within predetermined noise-induced cochlear pathology

**Figure 7:**
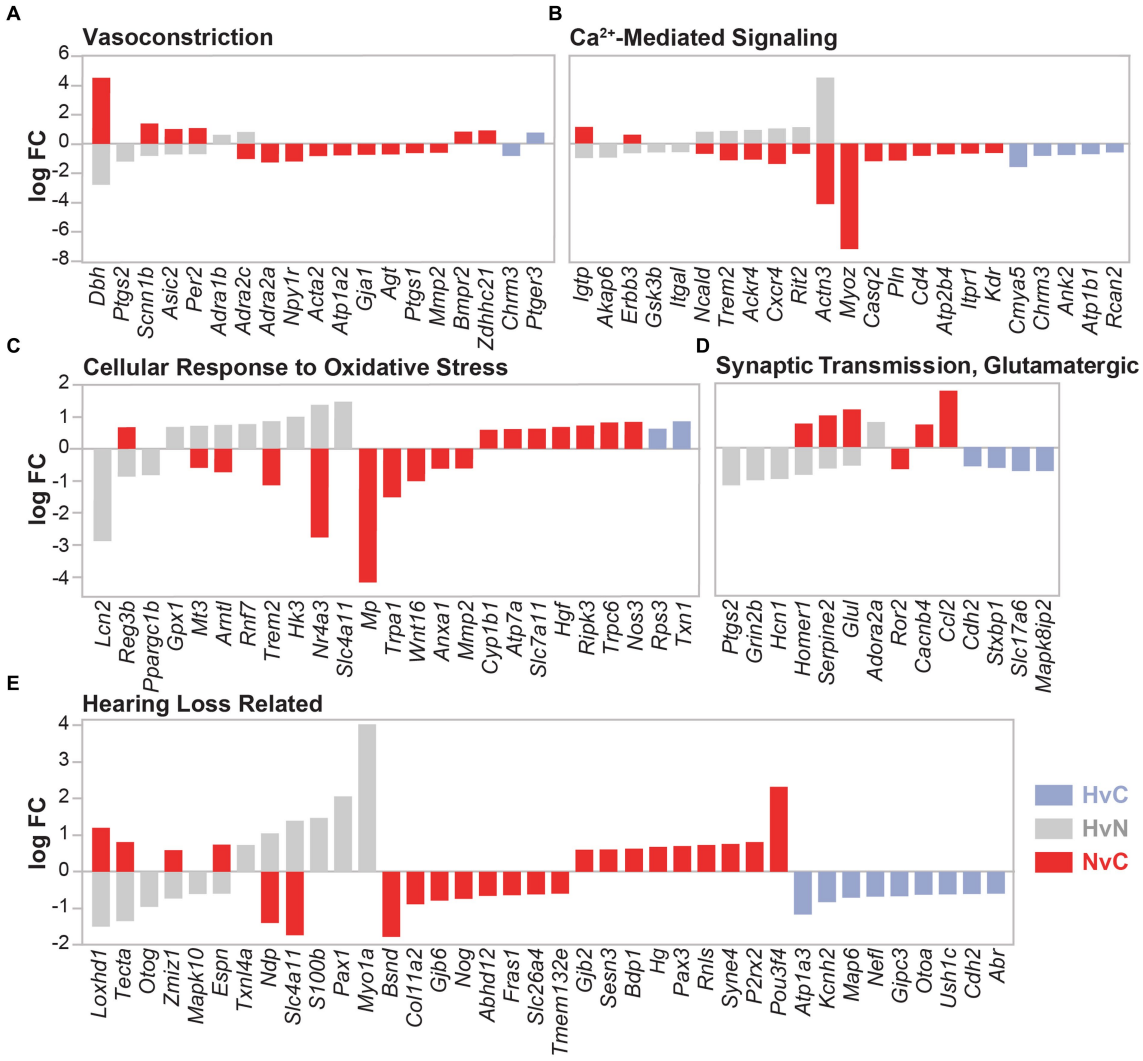
DEGs related to noise damage-associated pathways. DEG comparison of hypothermia versus normothermia (HvN, gray), normothermia versus control (NvC, red), and hypothermia versus control (HvC, light blue) is presented for several NIHL-associated pathologies including vasoconstriction **(A)**, calcium-mediated signaling **(B)**, cellular response to oxidative stress **(C)**, glutamatergic synaptic transmission **(D)**, and non-specific hearing loss **(E)**. Relative fold change in the DEGs in the associated comparison is presented on the y-axis with negative values corresponding to downregulation and positive values corresponding to upregulation.

Differential gene expression was also analyzed in the context of known NIHL mechanisms, including vasoconstriction, calcium signaling, oxidative stress, and glutamate excitotoxicity (Figures 7A–D). DEGs from all three group comparisons were also evaluated for all categories using curated gene lists. Gene ontology lists were utilized for the categories of vasoconstriction (GO:0042310, 110 genes), calcium-mediated signaling (GO:0019722, 190 genes), cellular response to oxidative stress (GO:0034599, 285 genes), and glutamatergic synaptic transmission (GO:0035249, 129 genes). A comprehensive list of 114 hearing loss-related genes was compiled from previous studies, including a genetic screen of mouse mutants with abnormal auditory sensitivity (Ingham et al., 2019) and the Gene4HL database for genetic data in hearing loss (Huang et al., 2021). These gene comparisons indicate mixed expression patterns between all groups, with the lowest total number of dysregulated genes observed in the HvC comparison. Notably, DEGs involved in vasoconstriction indicated higher dysregulation within the NvC group than in other group comparisons (Figure 7A). These included several cold-induced vasoconstrictive a2-adrenoceptors, such as Adra2a and Adra2c. A compensatory dysregulation was observed in the HvN group compared with the NvC group for the vasoconstrictive Adra2c. This hypothermic compensation was also observed for vasoconstrictive Dbh, Scnn1b, Asic2, and Per2 expression. Finally, in the HvC comparison, only two DEGs, Chrm3 and Ptger3, were not dysregulated in the other group comparisons.

In the calcium-mediated signaling gene set (Figure 7B), 15 genes were dysregulated in the NvC comparison, whereas HvN comparison showed dysregulation in 11 genes, with hypothermic directional compensation observed for eight genes. HvN comparison saw upregulation of six DEGs that were downregulated with normothermia TTM. These DEGs include Ncald, Trem2, Ackr4, Cxcr4, Rit2, and Actn3. A downregulation with hypothermia compared to normothermia group was observed for Igtp, which encodes a gamma interferon-induced GTPase, and Gsk3b, a gene involved in calcium homeostasis. DEGs that were only dysregulated within the HvC group include Cmya5, Ank2, Atp1b1, Rcan2, and the previously mentioned Chrm3.

A higher dysregulation of genes related to cellular response to oxidative stress (Figure 7C) was identified within the NvC comparison compared to other groups. Of the 17 NvC dysregulated DEGs related to oxidative stress, there was a notable upregulation of Nos3, which encodes nitric oxide synthase 3 and leads to nitric oxide generation through calcium signaling. Other DEGs upregulated with normothermia include Reg3b, Cyp1b1, Atp7a, Hgf, Ripk3, Slc7a11, and Trpc6. The downregulated genes in the NvC comparison with roles in oxidative stress response include Mmp2, Nr4a3, and Anxa1. Hypothermia induction post-noise produced a compensatory dysregulation for the gene expression of Reg3b, Mt3, Arntl, Nr4a3, and the previously mentioned Trem2. Of note, Gpx1, glutathione peroxidase-1, showed an increase in expression only with hypothermic intervention after noise injury in the HvN comparison. The GPX1 enzyme is a powerful antioxidant that scavenges H2O2 using reduced glutathione. Finally, for the HvC comparison, only two genes were implicated in the cellular response to oxidative stress, including the upregulation of the ribosomal-related Rps3 and the antioxidant-related Txn1.

Regarding DEGs involved in glutamatergic synaptic transmission (Figure 7D), the highest dysregulation was observed between the HvN comparisons, where compensatory upregulation of Homer1, Serpine2, and Glul was observed compared to NvC DEGs. Of the remaining dysregulated DEGs in the HvN comparison, downregulation of Ptgs2, Grin2b, and Hcn1 was observed, while Adora2a was the only gene in this category to be upregulated with post-noise hypothermia. Of the genes uniquely dysregulated in the HvC comparison, only a downregulation of Cdh2, Stxbp1, Slc17a6, and Mapk8ip2 was observed. These genes are hypothesized to be involved in many aspects of synaptic transmission, including neurite extension and vesicle loading and fusion.

For the genes previously implicated in hearing loss (Figure 7E), the highest dysregulation was again observed in the NvC comparison. All group comparisons identified differential gene expression of genes involved in deafness or hearing abnormalities. Normothermia TTM upregulated 13 genes associated with hearing loss, including Loxhd1, Tecta, Zmiz1, Espn, Gjb2, Sesn3, Bdp1, Hg, Pax3, Rnls, Syne4, P2rx2, and Pou3f4. Several of these targets include genes encoding transcription factors (Zmiz1, Pax3, Pou3f4), extracellular matrix proteins (Tecta, Gjb2), and cytoskeletal and stereociliary proteins (Loxhd1, Espn, Syne4). Compensatory dysregulation with hypothermia was observed in Loxhd1, Tecta, Zmiz1, Espn, Ndp, and Slc4a11. In the HvC comparison, downregulation of Atp1a3, Kcnh2, Map6, Nefl, Gipc3, Otoa, Ush1c, Cdh2, and Abr was observed.

## 4 Discussion

This study evaluated transcriptomic changes in hypothermia- and normothermia-treated animals after TTS-inducing noise exposure. Following related study (Rincon Sabatino et al., 2023), the noise exposure paradigm elicited temporary changes in ABR hearing thresholds and possibly noise-induced synaptopathy. The non-invasive and non-pharmacological cochlear cooling induced immediately after noise resulted in a mitigation of threshold shifts at 23 h post-noise. Cochleae extracted from these animals at 24 h post-noise were used to determine differential gene expression between the noise-exposed groups and compared to unexposed age-matched animals (Figure 1A).

### 4.1 TTS-inducing noise exposure is characterized by activation of inflammatory pathways

The first 12 to 48 h following acoustic injury are heavily associated with inflammation with increased expression of cytokines, including chemokines (CCs, CXCs), interferons (IFNs), interleukins (ILs), and tumor necrosis factors (TNFs; Kirkegaard et al., 2006; Patel et al., 2013; Cai et al., 2014; Yang et al., 2016; Maeda et al., 2017, 2018; Dhukhwa et al., 2019; Shin et al., 2019; Wang Y. et al., 2020; Wang Q. et al., 2020; Bae et al., 2021; Milon et al., 2021). These proinflammatory cytokines and adhesion molecules signal the activation of resident immune cells and recruitment of immune infiltrates involved in reparative processes after injury (Hirose et al., 2005; Yang et al., 2015; Frye et al., 2018). In this study, TTS-inducing exposure in the normothermia animals revealed a significant upregulation of genes for proinflammatory cytokines, including chemokines Cxcl10, Cxcl11, Cxcl9, and Ccl2, interleukin-associated Il6st and Il18bp, and several TNF family members, Tnfrsf1a, Tnfrsf11b, Tnfrsf12a, and Tnfrsf26. This comparison revealed that all upregulated biological processes after noise included DEGs involved in defense response. A significant upregulation of cellular response to interferon-beta (FDR = 1.23E-07) included regulators, such as Ifit3, Ifi47, Igtp, and Gbp1, involved in previously identified interferon signaling in PTS exposure models (Yang et al., 2015; Shin et al., 2022).

Interestingly, a distinct upregulation of complement activation was also observed in the TTS-induced injury involving genes, such as C1qb, C1r, C1s, C2, C4b, C4a, C6, Cfb, Clu, and Serping1. The most significantly upregulated transcript in the NvC animals was C4b or complement component 4 (logFC = 2.07), which has been previously shown to be upregulated in cochleae of aged C57BL/6 mice (Su et al., 2020). The complement system is known to modulate local inflammatory response and play a central role in the adaptive and innate immune response (Ricklin et al., 2010; Killick et al., 2018). Complement system activation and innate immunity have also been previously reported in response to PTS-inducing noise, including key regulators, such as C1s, C2, C6, and Cfb (Patel et al., 2013; Yang et al., 2016). The complement and coagulation cascade (FDR = 0.0086) was one of the most significant upregulated KEGG pathways observed in normothermia animals. These results suggest that the complement system is activated in the recovery of this TTS model, as previously seen in the cochlea in response to PTS-inducing noise exposure and aging. The complement may be involved in synaptic phenotypes due to its role in synaptic pruning. Ultimately, the comparison of post-noise normothermia and unexposed control animals establishes a possible involvement of interferon and complement signaling cascades. However, as there are expected differences in early stress response gene expression for TTS-inducing and PTS-inducing noise exposure (Lomax et al., 2001), there may be further exploration of target pathways, such as Toll-like receptor, TNF, IL-17, NFKB, and Jak/STAT signaling pathways, that were not as enriched with this TTS model.

### 4.2 Post-noise hypothermia limits noise-induced inflammation at acute recovery phase

Compared with post-noise normothermia, post-noise cochlear mild hypothermia therapy to 33°C post-noise showed reduced levels of multiple important cytokines and chemokines, such as Cxcl9, Cxcl10, Cxcl11, Timp1, and Osmr. Our observations are consistent with the literature on cytokine regulation with hypothermic intervention in the central nervous system (Truettner et al., 2005; Dugan et al., 2020). Furthermore, in the cochlea, our hypothermic intervention produced a strong downregulation of DEGs with immune host defense-related roles that have been frequently associated with early stress response (Lcn2, Timp1, Kng2, Vmo1, Il17rb, Mx2, Hpx, and Csf2rb). Of note, Lcn2, Lipocalin 2, is a significant marker in inflammation and apoptosis with a commonly observed strong upregulation in hypoxia-reoxygenation injury (Rognlien et al., 2012; Asaf et al., 2023) and has been upregulated in rodent models of repetitive noise, acute noise, and impulse noise (Kirkegaard et al., 2006; Han et al., 2012; Patel et al., 2013; Yang et al., 2016; Milon et al., 2021). Downregulation with hypothermic intervention suggests protection from cochlear inflammation, which has been observed in ischemic injury (Santora et al., 2010). It was also important to note that Timp1, TIMP metallopeptidase inhibitor 1, was downregulated with hypothermia. It is an inflammatory mediator and regulator of matrix metalloproteinase (MMP), which is upregulated in the cochlea and the dorsal cochlear nucleus following noise (Kirkegaard et al., 2006; Hu et al., 2012; Patel et al., 2013; Wu et al., 2017; Manohar et al., 2019). Following noise exposure, the expression of MMP (MMP-2, MMP-9) and TIMP is increased in the cochlea, particularly in the stria vascularis, with attributable roles in noise-induced tight juncture damage in the blood–labyrinth barrier (Wu et al., 2017). PTS-inducing noise has been well documented to have TNF-mediated hair cell loss (Hu et al., 2009; Fuentes-Santamaría et al., 2017). In our observations, TNF death receptors, TNFrsf10b and TNFrsf1a, were significantly downregulated with post-noise cochlear cooling further highlighting protective effects of hypothermia. A mitigation of GTPase activity was also observed with cooling-induced downregulation of Gbp1, Gbp4, Ifi47, Igtp, Irgm, LOC103690086, MGC108823, Mx1, Mx2, and RGD1305184 that were upregulated in the Noise+Normothermia group as compared to unexposed control animals. GTPase activity and association with the MAPK pathway have been linked to cisplatin-induced injury (Dhukhwa et al., 2021) and response to acute noise (Chen et al., 2012; Yang et al., 2015). Overall, these results combined with the literature strongly suggest multiple inflammatory pathways as interventional targets for cooling-enabled neuroprotection in the inner ear post-noise.

### 4.3 Clinical applications

Given the primarily focused intervention in inflammation, combination therapy with antioxidants or vasodilators may improve hypothermic cochlear neuroprotection. The Pharos platform can be used to identify appropriate repurposable drugs based on transcriptomic targets. Improvements to the hypothermic procedure may also be explored by extending duration, limiting depth, or increasing the repetition of cooling treatment. These avenues could be explored for more variable and traumatic noise exposures.

### 4.4 Limitations

In addition to the smaller sample size, only one timepoint was investigated to observe the acute changes related to TTS suppression by hypothermic intervention, which may limit the observance of cooling-induced dysregulated processes. Cooling applied within the first few hours post-noise could affect early transcription factors, leading to reduced inflammation, or also affect the ionic cochlear environment, thereby limiting calcium and glutamate excitotoxic damage. Accordingly, differential gene expression studies at an earlier time may implicate other important pathways involved in temperature management, including pyroptosis, early stress response modulation, or excitotoxicity. Future investigations into the ionic distribution in the endolymph after noise may provide context for hypothermic protection. Additionally, only male animals were used in this study. We noted hypothermic benefit in both male and female noise-exposed animals (Rincon Sabatino et al., 2023), although future comparison of both sexes will be necessary to fully understand the mechanisms by which hypothermia provides a beneficial effect. Finally, in the companion manuscript, we discuss in detail the influence of anesthetic agents on peripheral and central auditory physiology, including ABR thresholds, and evaluate the neuroprotection of ketamine anesthesia without intracochlear hypothermia (Rincon Sabatino et al., 2023). Ketamine alone did not protect from acute loss of paired synapses and presynaptic ribbons observed with immunohistological assessment for 24 h, supporting the results of the present study that the hypothermia treatment, and not a secondary effect from anesthesia, was globally driving the recovery post-noise.

## 5 Conclusion

The data suggest multiple pathways for noise-induced injury and hypothermic protection post-noise. Noise-induced cochlear injury involves biomolecular changes within the organ of Corti, leading to increased inflammation and hair cell death or dysfunction. Hypothermic intervention modulates several defense mechanisms associated with acoustic overexposure. This study identified potential protective pathways that prevent noise exposure-related threshold shifts and can lead to a comprehensive understanding of the benefits of hypothermia. Furthermore, the results from the DEG analysis may indicate potential combination treatments that can synergistically enhance the therapeutic benefit of post-noise hypothermia.

## Data availability statement

The raw dataset presented in this article is published at https://www.umgear.org/p?s=7aa6ff16 and has been made publicly available. The analysis, methods and details supporting the conclusions of this article will be made available by the authors, without undue reservation.

## Ethics statement

The animal study was approved by University of Miami Institute for Animal Care and Use Committee. The study was conducted in accordance with the local legislation and institutional requirements.

## Author contributions

SRi: Conceptualization, Data curation, Formal analysis, Funding acquisition, Investigation, Methodology, Visualization, Writing – original draft. RS: Data curation, Formal analysis, Methodology, Writing – review & editing. AG: Formal analysis, Visualization, Writing – review & editing. WD: Writing – review & editing. CK: Investigation, Methodology, Resources, Writing – review & editing. SRa: Conceptualization, Data curation, Formal analysis, Funding acquisition, Investigation, Methodology, Project administration, Resources, Software, Supervision, Validation, Visualization, Writing – original draft, Writing – review & editing.

## Footnotes

^1^ https://david.ncifcrf.gov/
