# Supplementary material for: Transcriptional response to mild therapeutic hypothermia in noise-induced cochlear injury
Source: Front Neurosci. 2024 Jan 17;17:1296475. doi: 10.3389/fnins.2023.1296475 (PMC10827921; doi:10.3389/fnins.2023.1296475)
Supplement: Supplementary file 1 [file Data_Sheet_1.PDF]

Supplementary Material

1 Supplementary Data

Supplemental Table 1. Hypothermia vs Normothermia: Molecular Functions & Cellular Component

		Term	Count	%	FE	FDR
**CC**	DOWN	extracellular space	*42*	17.95	2.71	1.564E-06
		neuron projection	*18*	7.69	3.89	4.561E-04
		neuronal cell body	*17*	7.26	2.67	3.643E-02
	UP	extracellular space	*70*	18.57	2.83	1.067E-12
		extracellular exosome	*87*	23.08	1.75	1.797E-05
		mitochondrial proton-transporting ATP synthase complex	*7*	1.86	16.93	0.0002
		troponin complex	*5*	1.33	33.26	0.0006
		axon	*21*	5.57	3.10	0.0010
		mitochondrial inner membrane	*17*	4.51	2.89	0.0131
		proteinaceous extracellular matrix	*15*	3.98	3.16	0.0131
		extracellular region	*27*	7.16	2.01	0.0339
		extracellular matrix	*14*	3.71	2.93	0.0339
**MF**	DOWN	GTPase activity	*12*	5.13	4.97	0.0117
	UP	RAGE receptor binding	*5*	1.33	28.61	0.0083

Abbreviations: FE: Fold Enrichment, FDR: False Discovery Rate, CC: Cellular Component, MF: Molecular Function

Supplementary Table 1.

Gene Ontology: Molecular Functions and Cellular Components

FE: Fold Enrichment, FDR: False Discovery Rate, CC: Cellular Component, MF: Molecular Function

Supplemental Table 2. Normothermia vs. Control: Molecular Functions & Cellular Component

		Term	Count	%	FE	FDR
**CC**	DOWN	extracellular space	*97*	19.36	2.94	1.093E-19
		proteinaceous extracellular matrix	*30*	5.99	4.73	1.260E-09
		extracellular region	*48*	9.58	2.68	1.444E-07
		Z disc	*17*	3.39	5.80	2.572E-06
		extracellular exosome	*107*	21.36	1.61	1.953E-05
		troponin complex	*6*	1.20	29.94	2.415E-05
		myofibril	*9*	1.80	8.98	0.0002
		basement membrane	*12*	2.40	5.15	0.0007
		extrinsic component of external side of plasma membrane	*5*	1.00	24.95	0.0008
		anchored component of membrane	*9*	1.80	6.53	0.0018
		integral component of plasma membrane	*44*	8.78	1.86	0.0028
		cell surface	*32*	6.39	2.09	0.0041
		I band	*6*	1.20	10.41	0.0049
		extracellular matrix	*18*	3.59	2.83	0.0049
		myosin complex	*8*	1.60	6.26	0.0050
		sarcomere	*7*	1.40	7.55	0.0051
		sarcoplasmic reticulum	*7*	1.40	6.35	0.0126
		contractile fiber	*5*	1.00	10.50	0.0177
		M band	*5*	1.00	9.98	0.0206
		axon	*20*	3.99	2.21	0.0279
		lysosome	*15*	2.99	2.45	0.0477
		collagen trimer	*7*	1.40	4.66	0.0495
	UP	apical plasma membrane	*18*	6.23	3.11	0.0120
		membrane	*43*	14.88	1.84	0.0149
		neuronal cell body	*22*	7.61	2.37	0.0336

		extracellular space	*40*	13.84	1.74	0.0393
		apical part of cell	*9*	3.11	4.50	0.0396
		extracellular region	*26*	9.00	2.04	0.0396
**MF**	DOWN	calcium ion binding	*43*	8.58	2.50	0.0001
		structural constituent of muscle	*8*	1.60	13.47	0.0004
		actin binding	*21*	4.19	3.41	0.0008
		frizzled binding	*8*	1.60	8.74	0.0046
		heparin binding	*14*	2.79	3.88	0.0086
		titin binding	*5*	1.00	18.37	0.0111
	UP	GTPase activity	*14*	4.73	4.62	0.0051

Abbreviations: FE: Fold Enrichment, FDR: False Discovery Rate, CC: Cellular Component, MF: Molecular Function

Supplementary Table 2.

Gene Ontology: Molecular Functions and Cellular Components

FE: Fold Enrichment, FDR: False Discovery Rate, CC: Cellular Component, MF: Molecular Function

Supplemental Table 3. Hypothermia vs. Control: Molecular Functions & Cellular Component

		**Term**	**Count**	**%**	**FE**	**FDR**
**CC**	DOWN	synaptic vesicle	*13*	7.78	12.61	8.590E-08
		terminal bouton	*12*	7.19	12.51	2.738E-07
		myelin sheath	*13*	7.78	8.52	2.494E-06
		cell junction	*16*	9.58	4.88	4.170E-05
		axon	*15*	8.98	5.20	4.170E-05
		synapse	*13*	7.78	5.53	0.0001
		axon terminus	*8*	4.79	11.12	0.0002
		dendrite	*15*	8.98	3.90	0.0007
		neuronal cell body	*15*	8.98	3.48	0.0017
		synaptic vesicle membrane	*6*	3.59	12.73	0.0017

		postsynaptic density	*10*	5.99	5.26	0.0019
		presynaptic membrane	*6*	3.59	10.15	0.0043
		voltage-gated potassium channel complex	*6*	3.59	9.88	0.0045
		intercalated disc	*5*	2.99	12.27	0.0087
		perikaryon	*7*	4.19	5.88	0.0140
		cytoskeleton	*8*	4.79	4.15	0.0340
	UP	cytosolic large ribosomal subunit	*24*	21.62	25.59	5.816E-24
		cytosolic small ribosomal subunit	*20*	18.02	34.27	2.711E-22
		ribosome	*20*	18.02	24.61	1.338E-19
		small ribosomal subunit	*9*	8.11	53.24	1.064E-10
		focal adhesion	*11*	9.91	5.09	0.0012
		membrane	*26*	23.42	2.17	0.0039
		extracellular exosome	*29*	26.13	2.01	0.0042
**MF**	DOWN	protein binding	*34*	20.36	2.78	1.937E-05
		voltage-gated potassium channel activity	*6*	3.59	14.45	0.0082
		protein kinase binding	*13*	7.78	3.86	0.0103
		delayed rectifier potassium channel activity	*5*	2.99	18.58	0.0103
	UP	structural constituent of ribosome	*42*	37.84	17.86	5.228E-39
		poly(A) RNA binding	*36*	32.43	5.68	4.069E-16
		RNA binding	*15*	13.51	5.78	1.277E-05

Abbreviations: FE: Fold Enrichment, FDR: False Discovery Rate, CC: Cellular Component, MF: Molecular Function

Supplementary Table 3.

Gene Ontology: Molecular Functions and Cellular Components

FE: Fold Enrichment, FDR: False Discovery Rate, CC: Cellular Component, MF: Molecular Function
